# Poly[[piperazine-1,4-dium [diaqua­tetra­kis­(μ-sulfanediyldiacetato)­dicerate(III)]] trihydrate]

**DOI:** 10.1107/S1600536811002923

**Published:** 2011-02-02

**Authors:** Mohammad Ghadermazi, Marilyn M. Olmstead, Shahideh Rostami, Jafar Attar Gharamaleki

**Affiliations:** a Department of Chemistry, Faculty of Science, University of Kurdistan, Sanandaj, Iran; b Department of Chemistry, One Shields Ave., University of California, Davis, CA, USA; c Faculty of Chemistry, Tarbiat Moallem University, Tehran, Iran

## Abstract

The title compound, (C_4_H_12_N_2_)[Ce_2_(C_4_H_4_O_4_S)_4_(H_2_O)_2_]·3H_2_O, features a polymeric anion with a centrosymmetric Ce_2_O_2_ core and a Ce⋯Ce distance of 4.3625 (4) Å. The anions form ribbons {[Ce_2_(C_4_H_4_O_4_S)_4_(H_2_O)_2_]^2−^}_*n*_ extending along [100]. The doubly protonated piperazinium cations reside on centers of inversion and link the polymeric ribbons *via* N—H⋯O hydrogen bonding. Each Ce^III^ cation is ten-coordinated by an O_2_S donor set from two tridentate sulfanediyldiacetate (tda) ligands, one water mol­ecule and three other tda O donors from adjacent {Ce(tda)_2_(H_2_O)} units in a distorted bicapped cubic environment. Additional O—H⋯O hydrogen bonding involving the coordinated and solvent water mol­ecules is also present. H atoms of the crystal water molecules could not be located and were not included in the refinement.

## Related literature

For the structure determination of a bis-sulfane­diyl­di­acetato­nickelate(II), see: Delaunay *et al.* (1976 ▶). For a dinuclear sulfanediyldiacetato complex, see: Baggio *et al.* (1999 ▶). For an example with a solely bidentate coordination mode of the sulfanediyldiacetato ligand, see: Marek *et al.* (2003 ▶). For bond-valence-sum calculations, see: Zhang *et al.* (2004 ▶).
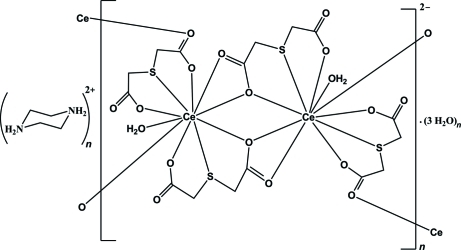


## Experimental

### 

#### Crystal data

(C_4_H_12_N_2_)[Ce_2_(C_4_H_4_O_4_S)_4_(H_2_O)_2_]·3H_2_O
*M* *_r_* = 1051.00
Triclinic, 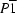

*a* = 6.4361 (7) Å
*b* = 11.1135 (12) Å
*c* = 12.5627 (14) Å
α = 96.693 (4)°
β = 104.646 (3)°
γ = 101.192 (3)°
*V* = 839.76 (16) Å^3^
*Z* = 1
Mo *K*α radiation
μ = 3.01 mm^−1^
*T* = 90 K
0.25 × 0.22 × 0.05 mm

#### Data collection

Bruker SMART APEXII diffractometer
Absorption correction: multi-scan (*SADABS*; Sheldrick, 1996 ▶) *T*_min_ = 0.520, *T*_max_ = 0.864
11816 measured reflections
4482 independent reflections
4433 reflections with *I* > 2σ(*I*)
*R*_int_ = 0.021

#### Refinement

*R*[*F* ^2^ > 2σ(*F* ^2^)] = 0.021
*wR*(*F* ^2^) = 0.056
*S* = 1.11
4482 reflections
229 parameters
3 restraints
H atoms treated by a mixture of independent and constrained refinement
Δρ_max_ = 1.79 e Å^−3^
Δρ_min_ = −1.81 e Å^−3^

### 

Data collection: *APEX2* (Bruker, 2009 ▶); cell refinement: *SAINT* (Bruker, 2009 ▶); data reduction: *SAINT*; program(s) used to solve structure: *SHELXS97* (Sheldrick, 2008 ▶); program(s) used to refine structure: *SHELXL97* (Sheldrick, 2008 ▶); molecular graphics: *XP* in *SHELXTL* (Sheldrick, 2008 ▶); software used to prepare material for publication: *SHELXL97*.

## Supplementary Material

Crystal structure: contains datablocks I, global. DOI: 10.1107/S1600536811002923/wm2442sup1.cif

Structure factors: contains datablocks I. DOI: 10.1107/S1600536811002923/wm2442Isup2.hkl

Additional supplementary materials:  crystallographic information; 3D view; checkCIF report

## Figures and Tables

**Table 1:** Selected bond lengths (Å)

Ce1—S1	3.2903 (6)
Ce1—S2	3.1445 (6)
Ce1—O1	2.5359 (15)
Ce1—O4	2.5069 (14)
Ce1—O5	2.4278 (14)
Ce1—O7	2.5117 (15)
Ce1—O8^i^	2.5024 (15)
Ce1—O3^ii^	2.6137 (16)
Ce1—O4^ii^	2.6542 (15)
Ce1—O9	2.6644 (15)

Symmetry codes: (i) 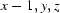; (ii) 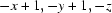.

**Table 2:** Hydrogen-bond geometry (Å, °)

*D*—H⋯*A*	*D*—H	H⋯*A*	*D*⋯*A*	*D*—H⋯*A*
O9—H9*C*⋯O7^i^	0.82 (2)	2.00 (2)	2.793 (2)	163 (3)
O9—H9*D*⋯O1^ii^	0.83 (2)	1.92 (2)	2.729 (2)	167 (3)
N1—H1*A*⋯O6^iii^	0.92	1.84	2.732 (2)	162
N1—H1*B*⋯O9^iv^	0.92	2.10	2.988 (2)	161

Symmetry codes: (i) 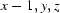; (ii) 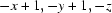; (iii) 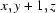; (iv) 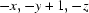.

**Fig. 1.**
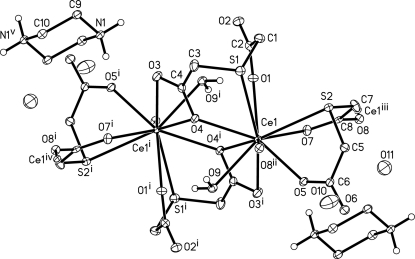
Thermal ellipsoid plot of the title compound at the 50% probability level. Hydrogen atoms are removed for clarity except those bonded to the piperazine N atoms and the coordinated water molecule. Hydrogen atoms are drawn as spheres of arbitrary radius. Atoms that indicate the propagation of the coordination polymer are shown. Symmetry codes: i = 1 - x, 1 - y, -z; ii = x - 1, y z; iii = 1 + x, y, z; iv = -x, 1 - y, -z; v = -x, 2 - y, -z.

**Fig. 2.**
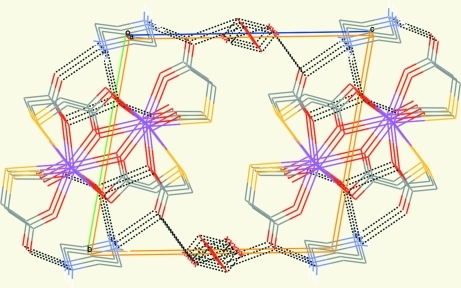
A view down [100] of the polymeric structure.

## supplementary crystallographic information

### Comment

Thiodiacetic acid is one class of dicarboxylic acid ligands that has been used for construction of coordination polymers. It is a versatile complexing agent with one sulfur and two oxygen donor atoms and can strongly complex metal ions. Although the structural study of sulfanediyldiacetate-transition metal compounds was initiated several decades ago (Delaunay, *et al.*, 1976), interest in the structural aspects of sulfanediyldiacetate compounds has remarkably increased in recent years, and many structures with *d*- and *f*-block metals are known to date. The ligand is usually tridentate, but at least one Mn(II) complex has been reported in which it is solely bidentate where the thioether S atom is not involved in bonding to the metal (Marek *et al.*, 2003). The ligand can be simply chelating or is involved in both bridging and chelating modes to give rise to dinuclear complexes (Baggio *et al.*, 1999).

The crystal structure of {(C_4_H_12_N_2_)[Ce(C_4_H_4_O_4_S)_2_(H_2_O)]_2_^.^3H_2_O}_n_ or {[(pipzH_2_)[Ce(tda)_2_(H_2_O)]_2_.3H_2_O}_n_, where tda = [S(CH_2_COO)_2_]^2-^, sulfanediyldiacetate, and pipzH_2_ is doubly protonated piperazine, is composed of a polymeric dinuclear anion [Ce(tda)_2_(H_2_O)]_2_^2-^, [pipzH_2_]^2+^ cations, and three water molecules of hydration. The components of the structure are shown in Fig. 1. The sulfanediyldiacetate group involving S1 behaves as both a tridentate chelating ligand and a bridging ligand to form a centrosymmetric dimer. The Ce1···Ce1^i^ (i = 1 - *x*, 1 - *y*, -*z*) distance is 4.3625 (4) Å. The sulfanediyldiacetate ligand involving S2 is also a tridentate chelating ligand while its oxygen, O8, coordinates to the Ce of an adjoining dimer and propagates the structure as a coordination polymer parallel to [100] (Fig. 2). The more distant Ce1^iii^ (iii = 1 + *x*, *y*, *z*) is 6.4361 (7) Å away from Ce1. The local coordination of the Ce^III^ cations consists of two thioethers (S1 and S2) and four O atoms (O1, O4, O5 and O7) from two chelating sulfanediyldiacetate groups, three oxygen atoms (O3, O4 and O8) of the carboxylate moieties from other sulfanediyldiacetate groups, and one oxygen atom from the coordinated water molecule (O9), resulting in an S_2_O_8_ distorted bicapped cubic environment. The Ce—O and Ce—S distances are normal and are gathered in Table 1. Bond valence sum calculations (Zhang *et al.*, 2004) yield a value of 2.9, in agreement with the oxidation state +III for the cerium atom. The [pipzH_2_]^2+^ cations and water molecules are further engaged in hydrogen bonding between polymeric units (Table 2). Although the H atoms of the uncoordinated water molecules could not be located, O···O contacts between 2.58 and 2.86 Å suggest that these molecules also participate in O—H···O hydrogen bonding.

### Experimental

The title compound was prepared by mixing two solutions containing 1.5 g (10 mmol) of 2,2'-thiodiacetic acid in 10 ml THF and 0.86 g (10 mmol) piperazine in 10 ml THF. A white precipitate was obtained after evaporating the solvent. An aqueous solution containing 0.34 g (1.5 mmol) of the obtained ion pair in 20 ml water was added dropwise to 0.21 g (0.5 mmol) Ce(NO_3_)_3_^.^6H_2_O in 15 ml water. After 60 min stirring and heating to 303 K, the solution became clear. Yellow crystals of the title compound were obtained after allowing the mixture to stand for 3 weeks at room temperature to evaporate the solvent.

### Refinement

The C-bound and N-bound hydrogen atoms were placed at calculated positions (C—H 0.99 Å, N—H 0.92 Å) and were treated as riding on their parent atoms with *U*(H) set to 1.2 *U*_eq_(C). The hydrogen atoms bonded to the coordinated water were located in a difference Fourier map and were refined with distance restraints of O—H 0.83 (2) Å and H···H 1.34 (4) Å and their isotropic displacement parameters allowed to refine. There are two sites for water moleculess of hydration. One of the hydrate molecules is disordered with respect to a center of symmetry and was kept at 0.5 occupancy and refined with an isotropic displacement parameter. Hydrogen atoms bonded to the water molecules of crystallisation could not be reliably located and were eventually omitted from the refinement. The highest peak in the final difference map is 0.83 Å from Ce1 and the largest hole is 0.87 Å from the same atom.

### Crystal data

**Table:**

(C_4_H_12_N_2_)[Ce_2_(C_4_H_4_O_4_S)_4_(H_2_O)_2_]·3H_2_O	*Z* = 1
*M**_r_* = 1051.00	*F*(000) = 520
Triclinic, *P*1	*D*_x_ = 2.078 Mg m^−^^3^
Hall symbol: -P 1	Mo *K*α radiation, λ = 0.71073 Å
*a* = 6.4361 (7) Å	Cell parameters from 9880 reflections
*b* = 11.1135 (12) Å	θ = 2.7–31.5°
*c* = 12.5627 (14) Å	µ = 3.01 mm^−^^1^
α = 96.693 (4)°	*T* = 90 K
β = 104.646 (3)°	Plate, colourless
γ = 101.192 (3)°	0.25 × 0.22 × 0.05 mm
*V* = 839.76 (16) Å^3^	

### Data collection

**Table:**

Bruker SMART APEXII diffractometer	4482 independent reflections
Radiation source: fine-focus sealed tube	4433 reflections with *I* > 2σ(*I*)
graphite	*R*_int_ = 0.021
Detector resolution: 8.3 pixels mm^-1^	θ_max_ = 29.1°, θ_min_ = 2.8°
ω scans	*h* = −8→8
Absorption correction: multi-scan (*SADABS*; Sheldrick, 1996)	*k* = −15→15
*T*_min_ = 0.520, *T*_max_ = 0.864	*l* = −17→17
11816 measured reflections	

### Refinement

**Table:**

Refinement on *F*^2^	Primary atom site location: structure-invariant direct methods
Least-squares matrix: full	Secondary atom site location: difference Fourier map
*R*[*F*^2^ > 2σ(*F*^2^)] = 0.021	Hydrogen site location: inferred from neighbouring sites
*wR*(*F*^2^) = 0.056	H atoms treated by a mixture of independent and constrained refinement
*S* = 1.11	*w* = 1/[σ^2^(*F*_o_^2^) + (0.0319*P*)^2^ + 0.9355*P*] where *P* = (*F*_o_^2^ + 2*F*_c_^2^)/3
4482 reflections	(Δ/σ)_max_ = 0.002
229 parameters	Δρ_max_ = 1.79 e Å^−^^3^
3 restraints	Δρ_min_ = −1.81 e Å^−^^3^

### Special details

**Table:**

Geometry. All e.s.d.'s (except the e.s.d. in the dihedral angle between two l.s. planes) are estimated using the full covariance matrix. The cell e.s.d.'s are taken into account individually in the estimation of e.s.d.'s in distances, angles and torsion angles; correlations between e.s.d.'s in cell parameters are only used when they are defined by crystal symmetry. An approximate (isotropic) treatment of cell e.s.d.'s is used for estimating e.s.d.'s involving l.s. planes.
Refinement. Refinement of *F*^2^ against ALL reflections. The weighted *R*-factor *wR* and goodness of fit *S* are based on *F*^2^, conventional *R*-factors *R* are based on *F*, with *F* set to zero for negative *F*^2^. The threshold expression of *F*^2^ > σ(*F*^2^) is used only for calculating *R*-factors(gt) *etc*. and is not relevant to the choice of reflections for refinement. *R*-factors based on *F*^2^ are statistically about twice as large as those based on *F*, and *R*- factors based on ALL data will be even larger.

### Fractional atomic coordinates and isotropic or equivalent isotropic displacement parameters (Å^2^)

**Table:**

	*x*	*y*	*z*	*U*_iso_*/*U*_eq_	Occ. (<1)
Ce1	0.572270 (15)	0.396056 (9)	0.136487 (8)	0.00772 (4)	
S1	0.50618 (8)	0.61593 (5)	0.31570 (4)	0.01299 (10)	
S2	0.81761 (8)	0.37549 (4)	0.38182 (4)	0.01047 (9)	
O1	0.8397 (2)	0.60785 (14)	0.18806 (12)	0.0126 (3)	
O2	0.9817 (3)	0.81191 (14)	0.24661 (13)	0.0155 (3)	
O3	0.4131 (3)	0.75180 (14)	0.03785 (13)	0.0150 (3)	
O4	0.3790 (2)	0.55553 (13)	0.05890 (12)	0.0116 (3)	
O5	0.5613 (2)	0.18686 (13)	0.17676 (12)	0.0121 (3)	
O6	0.5126 (3)	0.02205 (15)	0.25991 (14)	0.0204 (3)	
O7	0.9607 (2)	0.36963 (14)	0.16697 (12)	0.0128 (3)	
O8	1.2946 (2)	0.33740 (14)	0.24070 (12)	0.0118 (3)	
O9	0.1631 (2)	0.30107 (14)	0.00472 (12)	0.0118 (3)	
H9C	0.092 (5)	0.329 (3)	0.043 (2)	0.024 (8)*	
H9D	0.146 (6)	0.332 (3)	−0.052 (2)	0.034 (9)*	
N1	0.1458 (3)	0.94944 (16)	0.08009 (15)	0.0121 (3)	
H1A	0.2761	0.9585	0.1349	0.015*	
H1B	0.0661	0.8687	0.0701	0.015*	
C1	0.7895 (4)	0.7018 (2)	0.35817 (17)	0.0144 (4)	
H1C	0.8004	0.7868	0.3965	0.017*	
H1D	0.8795	0.6601	0.4116	0.017*	
C2	0.8790 (3)	0.70996 (19)	0.25730 (16)	0.0117 (3)	
C3	0.3913 (4)	0.7073 (2)	0.21616 (18)	0.0174 (4)	
H3A	0.2354	0.7020	0.2151	0.021*	
H3B	0.4716	0.7955	0.2429	0.021*	
C4	0.3986 (3)	0.67031 (19)	0.09779 (16)	0.0114 (3)	
C5	0.6813 (4)	0.2163 (2)	0.37811 (18)	0.0164 (4)	
H5A	0.7899	0.1761	0.4223	0.020*	
H5B	0.5635	0.2172	0.4156	0.020*	
C6	0.5786 (3)	0.13566 (19)	0.26210 (17)	0.0126 (4)	
C7	1.0804 (3)	0.3580 (2)	0.36422 (17)	0.0159 (4)	
H7A	1.1962	0.4274	0.4151	0.019*	
H7B	1.1077	0.2799	0.3895	0.019*	
C8	1.1111 (3)	0.35473 (17)	0.24746 (16)	0.0100 (3)	
C9	0.0173 (3)	1.03697 (19)	0.11667 (17)	0.0134 (4)	
H9A	0.1071	1.1235	0.1328	0.016*	
H9B	−0.0169	1.0173	0.1861	0.016*	
C10	−0.1952 (3)	1.02600 (19)	0.02648 (17)	0.0133 (4)	
H10A	−0.2903	0.9413	0.0146	0.016*	
H10B	−0.2757	1.0867	0.0502	0.016*	
O10	0.0974 (4)	0.0278 (2)	0.41702 (19)	0.0388 (5)	
O11	0.4926 (7)	0.0607 (4)	0.5455 (3)	0.0312 (8)*	0.50

### Atomic displacement parameters (Å^2^)

**Table:**

	*U*^11^	*U*^22^	*U*^33^	*U*^12^	*U*^13^	*U*^23^
Ce1	0.00901 (6)	0.00557 (6)	0.00889 (6)	0.00190 (4)	0.00262 (4)	0.00197 (4)
S1	0.0153 (2)	0.0122 (2)	0.0120 (2)	0.00179 (17)	0.00535 (18)	0.00315 (17)
S2	0.0117 (2)	0.0087 (2)	0.0111 (2)	0.00170 (16)	0.00410 (16)	0.00140 (16)
O1	0.0135 (7)	0.0103 (6)	0.0125 (6)	0.0009 (5)	0.0036 (5)	−0.0009 (5)
O2	0.0177 (7)	0.0109 (7)	0.0174 (7)	0.0011 (5)	0.0058 (6)	0.0022 (5)
O3	0.0214 (7)	0.0108 (7)	0.0148 (7)	0.0063 (6)	0.0060 (6)	0.0038 (5)
O4	0.0133 (6)	0.0093 (6)	0.0130 (6)	0.0045 (5)	0.0034 (5)	0.0028 (5)
O5	0.0146 (7)	0.0089 (6)	0.0132 (6)	0.0033 (5)	0.0038 (5)	0.0032 (5)
O6	0.0244 (8)	0.0098 (7)	0.0199 (8)	−0.0015 (6)	−0.0035 (6)	0.0060 (6)
O7	0.0130 (6)	0.0150 (7)	0.0124 (6)	0.0052 (5)	0.0045 (5)	0.0045 (5)
O8	0.0112 (6)	0.0116 (6)	0.0135 (6)	0.0029 (5)	0.0045 (5)	0.0030 (5)
O9	0.0143 (7)	0.0103 (6)	0.0115 (6)	0.0027 (5)	0.0048 (5)	0.0023 (5)
N1	0.0129 (8)	0.0080 (7)	0.0144 (8)	0.0024 (6)	0.0016 (6)	0.0027 (6)
C1	0.0165 (9)	0.0130 (9)	0.0118 (9)	0.0006 (7)	0.0033 (7)	0.0007 (7)
C2	0.0108 (8)	0.0120 (9)	0.0112 (8)	0.0022 (7)	0.0018 (7)	0.0015 (7)
C3	0.0234 (10)	0.0202 (10)	0.0123 (9)	0.0137 (8)	0.0049 (8)	0.0025 (7)
C4	0.0103 (8)	0.0123 (9)	0.0118 (8)	0.0047 (7)	0.0020 (7)	0.0021 (7)
C5	0.0209 (10)	0.0105 (9)	0.0132 (9)	−0.0015 (7)	−0.0010 (8)	0.0056 (7)
C6	0.0114 (8)	0.0096 (8)	0.0160 (9)	0.0026 (7)	0.0015 (7)	0.0036 (7)
C7	0.0112 (9)	0.0255 (11)	0.0114 (9)	0.0052 (8)	0.0033 (7)	0.0031 (8)
C8	0.0123 (8)	0.0048 (8)	0.0128 (8)	0.0011 (6)	0.0041 (7)	0.0010 (6)
C9	0.0172 (9)	0.0098 (8)	0.0132 (9)	0.0041 (7)	0.0037 (7)	0.0014 (7)
C10	0.0144 (9)	0.0114 (9)	0.0154 (9)	0.0039 (7)	0.0055 (7)	0.0027 (7)
O10	0.0566 (14)	0.0248 (10)	0.0376 (11)	0.0073 (9)	0.0213 (10)	0.0010 (8)

### Geometric parameters (Å, °)

**Table:**

Ce1—S1	3.2903 (6)	O9—H9D	0.825 (18)
Ce1—S2	3.1445 (6)	N1—C10^iii^	1.495 (3)
Ce1—O1	2.5359 (15)	N1—C9	1.497 (3)
Ce1—O4	2.5069 (14)	N1—H1A	0.9200
Ce1—O5	2.4278 (14)	N1—H1B	0.9200
Ce1—O7	2.5117 (15)	C1—C2	1.524 (3)
Ce1—O8^i^	2.5024 (15)	C1—H1C	0.9900
Ce1—O3^ii^	2.6137 (16)	C1—H1D	0.9900
Ce1—O4^ii^	2.6542 (15)	C3—C4	1.511 (3)
Ce1—O9	2.6644 (15)	C3—H3A	0.9900
S1—C1	1.798 (2)	C3—H3B	0.9900
S1—C3	1.806 (2)	C5—C6	1.531 (3)
S2—C7	1.804 (2)	C5—H5A	0.9900
S2—C5	1.805 (2)	C5—H5B	0.9900
O1—C2	1.287 (2)	C7—C8	1.526 (3)
O2—C2	1.236 (3)	C7—H7A	0.9900
O3—C4	1.248 (2)	C7—H7B	0.9900
O4—C4	1.282 (2)	C9—C10	1.513 (3)
O5—C6	1.262 (2)	C9—H9A	0.9900
O6—C6	1.246 (3)	C9—H9B	0.9900
O7—C8	1.262 (2)	C10—H10A	0.9900
O8—C8	1.255 (2)	C10—H10B	0.9900
O9—H9C	0.817 (17)		
			
O5—Ce1—O8^i^	69.44 (5)	C8—O7—Ce1	136.54 (13)
O5—Ce1—O4	150.35 (5)	C8—O8—Ce1^iv^	140.41 (13)
O8^i^—Ce1—O4	91.13 (5)	Ce1—O9—H9C	101 (2)
O5—Ce1—O7	74.48 (5)	Ce1—O9—H9D	109 (3)
O8^i^—Ce1—O7	131.20 (5)	H9C—O9—H9D	108 (3)
O4—Ce1—O7	133.20 (5)	C10^iii^—N1—C9	111.17 (15)
O5—Ce1—O1	138.75 (5)	C10^iii^—N1—H1A	109.4
O8^i^—Ce1—O1	121.49 (5)	C9—N1—H1A	109.4
O4—Ce1—O1	70.37 (5)	C10^iii^—N1—H1B	109.4
O7—Ce1—O1	70.20 (5)	C9—N1—H1B	109.4
O5—Ce1—O3^ii^	69.93 (5)	H1A—N1—H1B	108.0
O8^i^—Ce1—O3^ii^	121.93 (5)	C2—C1—S1	110.59 (14)
O4—Ce1—O3^ii^	105.26 (5)	C2—C1—H1C	109.5
O7—Ce1—O3^ii^	71.75 (5)	S1—C1—H1C	109.5
O1—Ce1—O3^ii^	116.48 (5)	C2—C1—H1D	109.5
O5—Ce1—O4^ii^	119.23 (5)	S1—C1—H1D	109.5
O8^i^—Ce1—O4^ii^	144.10 (5)	H1C—C1—H1D	108.1
O4—Ce1—O4^ii^	64.63 (6)	O2—C2—O1	124.50 (19)
O7—Ce1—O4^ii^	82.62 (5)	O2—C2—C1	119.06 (18)
O1—Ce1—O4^ii^	76.75 (5)	O1—C2—C1	116.43 (18)
O3^ii^—Ce1—O4^ii^	49.44 (5)	C4—C3—S1	115.90 (15)
O5—Ce1—O9	85.38 (5)	C4—C3—H3A	108.3
O8^i^—Ce1—O9	66.23 (5)	S1—C3—H3A	108.3
O4—Ce1—O9	65.95 (5)	C4—C3—H3B	108.3
O7—Ce1—O9	141.72 (5)	S1—C3—H3B	108.3
O1—Ce1—O9	135.87 (5)	H3A—C3—H3B	107.4
O3^ii^—Ce1—O9	70.84 (5)	O3—C4—O4	121.12 (19)
O4^ii^—Ce1—O9	79.35 (5)	O3—C4—C3	119.05 (19)
O5—Ce1—S2	63.64 (4)	O4—C4—C3	119.73 (18)
O8^i^—Ce1—S2	70.54 (4)	C6—C5—S2	116.14 (15)
O4—Ce1—S2	132.01 (3)	C6—C5—H5A	108.3
O7—Ce1—S2	64.42 (3)	S2—C5—H5A	108.3
O1—Ce1—S2	82.04 (4)	C6—C5—H5B	108.3
O3^ii^—Ce1—S2	122.24 (4)	S2—C5—H5B	108.3
O4^ii^—Ce1—S2	145.36 (3)	H5A—C5—H5B	107.4
O9—Ce1—S2	133.47 (3)	O6—C6—O5	124.6 (2)
C4^ii^—Ce1—S2	139.09 (4)	O6—C6—C5	116.06 (18)
O5—Ce1—S1	120.96 (4)	O5—C6—C5	119.27 (18)
O8^i^—Ce1—S1	62.01 (4)	C8—C7—S2	117.92 (15)
O4—Ce1—S1	62.43 (4)	C8—C7—H7A	107.8
O7—Ce1—S1	115.38 (4)	S2—C7—H7A	107.8
O1—Ce1—S1	60.23 (4)	C8—C7—H7B	107.8
O3^ii^—Ce1—S1	167.65 (3)	S2—C7—H7B	107.8
O4^ii^—Ce1—S1	119.75 (3)	H7A—C7—H7B	107.2
O9—Ce1—S1	102.88 (3)	O8—C8—O7	124.80 (19)
C4^ii^—Ce1—S1	144.33 (4)	O8—C8—C7	114.35 (17)
S2—Ce1—S1	69.898 (14)	O7—C8—C7	120.85 (18)
C1—S1—C3	99.14 (11)	N1—C9—C10	110.34 (16)
C1—S1—Ce1	95.72 (7)	N1—C9—H9A	109.6
C3—S1—Ce1	97.65 (7)	C10—C9—H9A	109.6
C7—S2—C5	101.63 (11)	N1—C9—H9B	109.6
C7—S2—Ce1	100.18 (7)	C10—C9—H9B	109.6
C5—S2—Ce1	97.24 (7)	H9A—C9—H9B	108.1
C2—O1—Ce1	138.15 (13)	N1^iii^—C10—C9	110.03 (17)
C4—O3—Ce1^ii^	95.97 (12)	N1^iii^—C10—H10A	109.7
C4—O4—Ce1	131.30 (13)	C9—C10—H10A	109.7
C4—O4—Ce1^ii^	93.20 (12)	N1^iii^—C10—H10B	109.7
Ce1—O4—Ce1^ii^	115.37 (5)	C9—C10—H10B	109.7
C6—O5—Ce1	136.68 (13)	H10A—C10—H10B	108.2
			
O5—Ce1—S1—C1	108.05 (8)	O1—Ce1—O4—Ce1^ii^	84.25 (6)
O8^i^—Ce1—S1—C1	146.53 (8)	O3^ii^—Ce1—O4—Ce1^ii^	−28.92 (7)
O4—Ce1—S1—C1	−105.85 (8)	O4^ii^—Ce1—O4—Ce1^ii^	0.0
O7—Ce1—S1—C1	21.53 (8)	O9—Ce1—O4—Ce1^ii^	−89.30 (6)
O1—Ce1—S1—C1	−23.78 (8)	S2—Ce1—O4—Ce1^ii^	142.97 (3)
O3^ii^—Ce1—S1—C1	−101.58 (18)	S1—Ce1—O4—Ce1^ii^	150.14 (7)
O4^ii^—Ce1—S1—C1	−74.64 (8)	O8^i^—Ce1—O5—C6	−53.69 (19)
O9—Ce1—S1—C1	−159.61 (8)	O4—Ce1—O5—C6	−105.6 (2)
S2—Ce1—S1—C1	68.49 (7)	O7—Ce1—O5—C6	92.87 (19)
O5—Ce1—S1—C3	−151.90 (9)	O1—Ce1—O5—C6	61.1 (2)
O8^i^—Ce1—S1—C3	−113.42 (9)	O3^ii^—Ce1—O5—C6	168.7 (2)
O4—Ce1—S1—C3	−5.79 (9)	O4^ii^—Ce1—O5—C6	164.92 (18)
O7—Ce1—S1—C3	121.59 (9)	O9—Ce1—O5—C6	−120.01 (19)
O1—Ce1—S1—C3	76.28 (9)	S2—Ce1—O5—C6	24.12 (18)
O3^ii^—Ce1—S1—C3	−1.53 (19)	S1—Ce1—O5—C6	−17.8 (2)
O4^ii^—Ce1—S1—C3	25.42 (9)	O5—Ce1—O7—C8	−65.42 (18)
O9—Ce1—S1—C3	−59.56 (9)	O8^i^—Ce1—O7—C8	−22.1 (2)
S2—Ce1—S1—C3	168.54 (8)	O4—Ce1—O7—C8	127.01 (18)
O5—Ce1—S2—C7	84.51 (9)	O1—Ce1—O7—C8	92.90 (19)
O8^i^—Ce1—S2—C7	160.59 (9)	O3^ii^—Ce1—O7—C8	−138.95 (19)
O4—Ce1—S2—C7	−126.28 (9)	O4^ii^—Ce1—O7—C8	171.42 (19)
O7—Ce1—S2—C7	−0.10 (9)	O9—Ce1—O7—C8	−126.28 (18)
O1—Ce1—S2—C7	−71.91 (9)	S2—Ce1—O7—C8	2.38 (17)
O3^ii^—Ce1—S2—C7	44.46 (9)	S1—Ce1—O7—C8	51.92 (19)
O4^ii^—Ce1—S2—C7	−19.46 (10)	C3—S1—C1—C2	−56.27 (16)
O9—Ce1—S2—C7	138.09 (9)	Ce1—S1—C1—C2	42.45 (15)
S1—Ce1—S2—C7	−133.04 (8)	Ce1—O1—C2—O2	−159.61 (15)
O5—Ce1—S2—C5	−18.75 (9)	Ce1—O1—C2—C1	20.0 (3)
O8^i^—Ce1—S2—C5	57.33 (9)	S1—C1—C2—O2	132.49 (17)
O4—Ce1—S2—C5	130.46 (9)	S1—C1—C2—O1	−47.1 (2)
O7—Ce1—S2—C5	−103.36 (9)	C1—S1—C3—C4	87.74 (18)
O1—Ce1—S2—C5	−175.16 (9)	Ce1—S1—C3—C4	−9.35 (18)
O3^ii^—Ce1—S2—C5	−58.80 (9)	Ce1^ii^—O3—C4—O4	−5.3 (2)
O4^ii^—Ce1—S2—C5	−122.72 (10)	Ce1^ii^—O3—C4—C3	178.13 (16)
O9—Ce1—S2—C5	34.83 (9)	Ce1—O4—C4—O3	133.58 (17)
S1—Ce1—S2—C5	123.70 (8)	Ce1^ii^—O4—C4—O3	5.2 (2)
O5—Ce1—O1—C2	−97.7 (2)	Ce1—O4—C4—C3	−49.9 (3)
O8^i^—Ce1—O1—C2	−3.5 (2)	Ce1^ii^—O4—C4—C3	−178.26 (17)
O4—Ce1—O1—C2	75.31 (19)	Ce1—O4—C4—Ce1^ii^	128.35 (15)
O7—Ce1—O1—C2	−130.4 (2)	S1—C3—C4—O3	−150.05 (17)
O3^ii^—Ce1—O1—C2	173.03 (18)	S1—C3—C4—O4	33.4 (3)
O4^ii^—Ce1—O1—C2	142.8 (2)	C7—S2—C5—C6	−79.97 (18)
O9—Ce1—O1—C2	83.8 (2)	Ce1—S2—C5—C6	22.04 (17)
S2—Ce1—O1—C2	−64.81 (19)	Ce1—O5—C6—O6	160.71 (16)
S1—Ce1—O1—C2	6.54 (18)	Ce1—O5—C6—C5	−17.5 (3)
O5—Ce1—O4—C4	135.03 (17)	S2—C5—C6—O6	170.16 (17)
O8^i^—Ce1—O4—C4	87.52 (17)	S2—C5—C6—O5	−11.5 (3)
O7—Ce1—O4—C4	−69.76 (19)	C5—S2—C7—C8	98.32 (18)
O1—Ce1—O4—C4	−35.69 (17)	Ce1—S2—C7—C8	−1.33 (17)
O3^ii^—Ce1—O4—C4	−148.85 (17)	Ce1^iv^—O8—C8—O7	32.5 (3)
O4^ii^—Ce1—O4—C4	−119.94 (19)	Ce1^iv^—O8—C8—C7	−146.95 (16)
O9—Ce1—O4—C4	150.77 (18)	Ce1—O7—C8—O8	176.41 (13)
S2—Ce1—O4—C4	23.04 (19)	Ce1—O7—C8—C7	−4.1 (3)
S1—Ce1—O4—C4	30.20 (16)	S2—C7—C8—O8	−177.31 (15)
O5—Ce1—O4—Ce1^ii^	−105.03 (10)	S2—C7—C8—O7	3.2 (3)
O8^i^—Ce1—O4—Ce1^ii^	−152.54 (6)	C10^iii^—N1—C9—C10	57.6 (2)
O7—Ce1—O4—Ce1^ii^	50.18 (9)	N1—C9—C10—N1^iii^	−57.0 (2)

Symmetry codes: (i) *x*−1, *y*, *z*; (ii) −*x*+1, −*y*+1, −*z*; (iii) −*x*, −*y*+2, −*z*; (iv) *x*+1, *y*, *z*.

### Hydrogen-bond geometry (Å, °)

**Table:**

*D*—H···*A*	*D*—H	H···*A*	*D*···*A*	*D*—H···*A*
O9—H9C···O7^i^	0.82 (2)	2.00 (2)	2.793 (2)	163 (3)
O9—H9D···O1^ii^	0.83 (2)	1.92 (2)	2.729 (2)	167 (3)
N1—H1A···O6^v^	0.92	1.84	2.732 (2)	162
N1—H1B···O9^vi^	0.92	2.10	2.988 (2)	161

Symmetry codes: (i) *x*−1, *y*, *z*; (ii) −*x*+1, −*y*+1, −*z*; (v) *x*, *y*+1, *z*; (vi) −*x*, −*y*+1, −*z*.
